# The effect of pasireotide on intestinal anastomotic healing with and without whole-body irradiation in a rat model

**DOI:** 10.1007/s00384-018-3193-5

**Published:** 2018-11-27

**Authors:** Gabriel J. Seifert, Gunnar Leithold, Birte Kulemann, Philipp A. Holzner, Torben Glatz, Jens Hoeppner, Simon Kirste, Goran Marjanovic, Claudia Laessle

**Affiliations:** 1 Department of General and Visceral Surgery, Medical Centre – University of Freiburg, Faculty of Medicine, University Freiburg, Hugstetter Str. 55, 79106 Freiburg, Germany; 2 Department of Radiooncology, Medical Centre – University of Freiburg, Faculty of Medicine, University Freiburg, Robert-Koch-Str. 3, 79106 Freiburg, Germany

**Keywords:** Anastomosis, Intestinal healing, Irradiation, SOM230, Pasireotide

## Abstract

**Objective:**

To examine pasireotide’s effect on intestinal anastomotic healing under physiological conditions and following preoperative whole-body irradiation.

**Material and methods:**

Forty-five male Wistar rats received an ileoileal end-to-end anastomosis. Group 1 (Co, *n* = 9) served as control. Group 2 (SOM, *n* = 10) received pasireotide (60 mg/kg) 6 days preoperatively. Group 3 (R-Co, *n* = 13) was subjected to 8 Gy whole-body irradiation 4 days preoperatively. Finally, group 4 (R-SOM, *n* = 13) received pasireotide 6 days preoperatively and whole-body irradiation 4 days preoperatively. On postoperative day 4, anastomotic bursting pressure, histology, IGF-1 staining, and collagen density were examined.

**Results:**

Mortality was higher in irradiated animals (30.8% vs. 5.3%, *p* = 0.021), and anastomotic bursting pressure was significantly lower (median, R-Co = 83 mmHg; R-SOM = 101 mmHg; Co = 149.5 mmHg; SOM = 169 mmHg). Inflammation measured by leukocyte infiltration following irradiation was reduced (*p* = 0.023), and less collagen was observed, though this was not statistically significant. Bursting pressure did not significantly differ between Co and SOM and between R-Co and R-SOM animals respectively. Semi-quantitative scoring of IGF-1, fibroblast bridging, or collagen density did not reveal significant differences among the groups.

**Conclusion:**

Whole-body irradiation decreases the quality of intestinal anastomotic wound healing and increases mortality. Pasireotide does not significantly lessen this detrimental effect.

## Introduction

Intestinal anastomotic healing is a complex process that involves intense genetic regulation of a large set of genes and the coordinated interaction of multiple cell types. Radiotherapy is applied preoperatively or postoperatively in different clinical scenarios and may interfere with anastomotic healing after surgery. Irradiation causes substantial gastro-intestinal toxicity and is known to have a detrimental effect on the vulnerable process of anastomotic healing. This can be quantified using various quality parameters of healing (e.g., bursting pressure, collagen density, histology, and leakage rate), thus rendering the anastomosis far more instable [3, 7, 10, 17, 19, 26].

Pasireotide, a somatostatine analogue with high somatostatine receptor affinity, is used to treat a spectrum of diseases ranging from acromegaly to Cushing’s disease and especially postoperative pancreatic fistula [4]. Application transiently and effectively decreases growth hormone (GH) and insulin-like growth factor 1 (IGF-1) levels across species [27]. Additionally, it drastically throttles exocrine pancreatic function [2]. Fu et al. demonstrated that pasireotide reduces mortality, mitigates intestinal injury, and largely prevents subsequent bacteraemia given before or shortly after whole-body irradiation (WBI) in mice. These effects were largely reversible upon substitution of pancreatic enzymes [9].

Although the reduction of pancreatic enzymes was shown to improve radiation-induced GI toxicity and may lead to better anastomotic healing, the other effect of pasireotide, a decrease of IGF-1 and GH levels, may lead to negative effects on anastomotic healing.

Based on these data, we wanted to investigate the role of pasireotide on anastomotic healing without and with neoadjuvant radiation therapy. However, various studies have convincingly suggested a positive effect of IGF-1 on the process of anastomotic healing. Furthermore, in a previous study, we observed a significant increase in local IGF-1 gene expression during intestinal anastomotic healing [22]. Therefore, it may not be excluded that pasireotide negatively impacts physiological anastomotic healing via decreasing systemic and/or local IGF-1 production.

To shed light on the influence of pasireotide on intestinal healing, we examined its effect under control conditions as well as following WBI irradiation in a rat model of intestinal anastomotic healing.

## Material and methods

### Animals and groups

The local Ethics Committee at the University of Freiburg and the local district government in Freiburg approved all experiments. All procedures were performed under strict adherence to the German Animal Welfare Law and met the standards set in the “Guide for care and use of laboratory animals” prepared by the National Academy of Sciences and published by the National Institutes of Health (NIH Publication No. 86-23, revised 1985). All applicable international, national, and institutional guidelines for the care and use of animals were followed. Forty-five male Wistar rats (Charles River, Sulzfeld, Germany) with a bodyweight of 220 to 380 g were randomly divided into four groups. All animals were laparotomized, and an ileoileal anastomosis was performed. The anastomosis was resected on postoperative day 4. Group 1 (Co, *n* = 9) served as a control group and received no additional perioperative treatment. Group 2 (SOM, *n* = 10) received a subcutaneous injection of 60 mg/kg pasireotide long-acting release 6 days prior to formation of anastomosis. This form of pasireotide is typically administered every 28 days. Due to initial modulation of blood glucose by pasireotide, an effect associated with the drug and validated in our trial, it was applied 2 days prior to irradiation treatment to avoid an overlap of effects. Furthermore, this time window was used by Fu et al. who demonstrated amelioration of irradiation effects [8]. Group 3 (R-Co, *n* = 13) was subjected to 8 Gy of WBI 4 days prior to formation of anastomosis. Finally, group 4 (R-SOM, *n* = 13) received a subcutaneous injection of 60 mg/kg pasireotide 6 days preoperatively and was subjected to 8 Gy of WBI 4 days prior to formation of anastomosis.

### Medication

Pasireotide long-acting reagent was supplied by Novartis. This application form has the advantage of only having to be administered once per month due to steady release. The drug was suspended in 2-ml carrier solution and administered subcutaneously. Blood glucose levels and bodyweight were regularly measured in the SOM group after injection, because an initial increase of blood glucose is indicative of successful administration.

### Irradiation

WBI was performed 4 days preoperatively in groups 3 (RTxCo) and 4 (RTxSOM) with a Cobalt 60-γ-irradiator (Philips) in one session. Animals were placed in a perforated custom-made Plexiglas box (20 × 15 × 8 cm). The box was placed in an irradiation area of 20 × 25 cm at a distance of 80 cm. A total dose of 8 Gy was applied at a dose rate of 0.278 Gy/min for 32 min. This dose has been shown to cause substantial intestinal and hematopoietic damage in mice [8].

### Perioperative setup and formation of anastomosis

All animals in this study received the same operation. Two animals were housed per cage (Ebeco, Castrop-Rauxel, Germany) on animal bedding (Ssniff, Soest, Germany), fed with standard chow (Ssniff; contents, 18,000 IU/kg vitamin A; 1.280 IU/kg vitamin D 3; 120 mg/kg vitamin E) and given access to tap water ad libitum. Twelve hours before anesthesia, they were deprived of food but had free access to water. After inducing narcosis in an induction chamber using 4% isoflurane, continuous anesthesia was sustained with 1.5% isoflurane by mask. Intraoperative fluid loss was replaced by a one-time subcutaneous injection of 9 ml/kg 0.9% crystalloid fluid. The operative procedure was performed as described previously [16]. In summary, a 1-cm ileal segment was resected approximately 15 cm proximal to the cecum. Ileal continuity was restored by creating an end-to-end anastomosis. All operations were performed by one surgeon. We used eight inverting interrupted sutures (Prolene 8/0 BVI30-5; Ethicon, Nordstedt, Germany) for the anastomosis with a distance of 1–2 mm between sutures.

In order to prevent obstruction of the anastomosis, a 2-cm silicone catheter with a diameter of 5 mm (Heidelberger Verlängerung; Braun, Melsungen, Germany) was introduced into the proximal and distal lumen of the resected bowel. After suturing the anterior wall, the catheter was removed before suturing the posterior wall.

### Re-laparotomy and macroscopic examination

Re-laparotomy was performed on postoperative day 4 (POD 4). Suture strength dramatically decreases during the first 3 postoperative days [14] whereas tissue strength of the anastomoses ought to increase. If this process is disrupted, early anastomotic insufficiency occurs, which can be detected after POD 3. Anastomotic insufficiencies are often associated with mortality in rodents. We therefore performed the investigation on POD 4.

After induction of anesthesia, animals were euthanized by cardiac puncture and potassium injection at a lethal dose of 10 mmol/kg. The abdomen was reopened with a complete midline and horizontal incision for optimal abdominal exposure. Signs of intra-abdominal inflammation, peritonitis, and intra-abdominal abscesses were documented. Intra-abdominal adhesions were noted and graded according to the following scoring system established by van der Ham [23]: 0 = no adhesion; 1 = adhesions to one structure; 2 = adhesions to two structures; and 3 = adhesions to three or more structures. Perianastomotic adhesions were not dissected. An approximately 4-cm segment containing the anastomosis was resected and carefully cleaned of fecal remnants.

### Measurement of bursting pressure

The anastomotic bursting pressure was the primary outcome measure and was determined as described previously [16]. The maximum pressure and the bursting site (anastomosis or distant from the anastomosis) were documented.

### Histological examination

Histological sections (2–3 μm) containing the suture line in the middle were stained with hematoxylin-eosin, sirius red, and alcian blue. Additionally, immunohistochemical staining for IGF-1 was performed (clone M23 mouse monoclonal antibody; Thermo Scientific). All histological sections were examined in a blinded manner by two pathologists in a semi-quantitative fashion adapted from Biert et al. [3]. IGF-1 staining was scored according to Guven (0 = no staining, 1 = weak staining, 2 = moderate staining, and 3 = strong staining (> 50% of cells)) [11].

### Statistics

All data were analyzed using SPSS for Microsoft Windows (IBM Corp. Released 2011. IBM SPSS Statistics for Windows, Version 20.0. Armonk, NY, IBM Corp.). To describe the bursting pressure, the median value is shown. For all other data, mean values with standard deviations are depicted. The chi-square test and Fisher’s exact test were used to describe differences in frequency distribution. The *U* test according to Mann and Whitney and the paired *t* test were used to test for statistical significance. Correlations were assessed using the Spearman correlation coefficient. Significance was defined at 5% (*p* = 0.05).

## Results

### Bodyweight and blood glucose levels following SOM injection

**Fig. 1:**
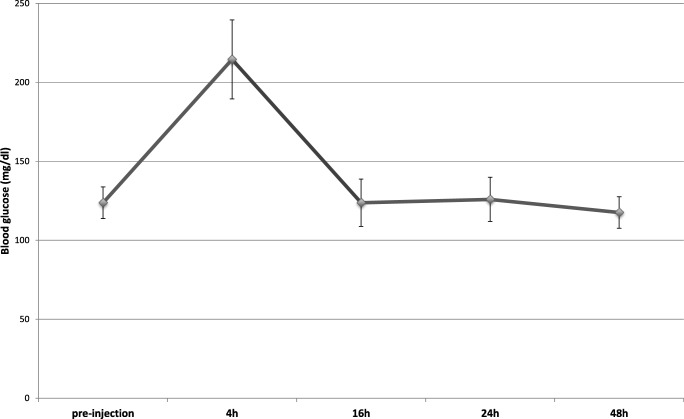
Mean blood glucose levels following pasireotide injections (± SD) in the SOM group. After initial increase of glucose level, homeostasis is restored 16 h after injection

Administering 60 mg/kg pasireotide did not lead to morbidity in either group. In the animals of the SOM group, we observed a mean loss of bodyweight of 9.1 g from injection to postoperative day 4. However, this was not significant. To check for successful application of pasireotide, we measured blood glucose levels prior to injection and during the next 48 h in blood aspirated from the tail (Fig. 1). After an initial peak of blood glucose levels in all animals (*p* < 0.001), values normalized after 16 h (*p* < 0.001) confirming successful application.

### Bodyweight following irradiation

Exposure to WBI induced a subtle loss of fur and relevant weight loss during the 4 days between irradiation and laparotomy with a mean loss of 38.9 g in R-Co animals and 41.9 g in R-SOM animals. However, this difference was not statistically significant (*p* > 0.05).

### Mortality and causes of death

**Fig. 2:**
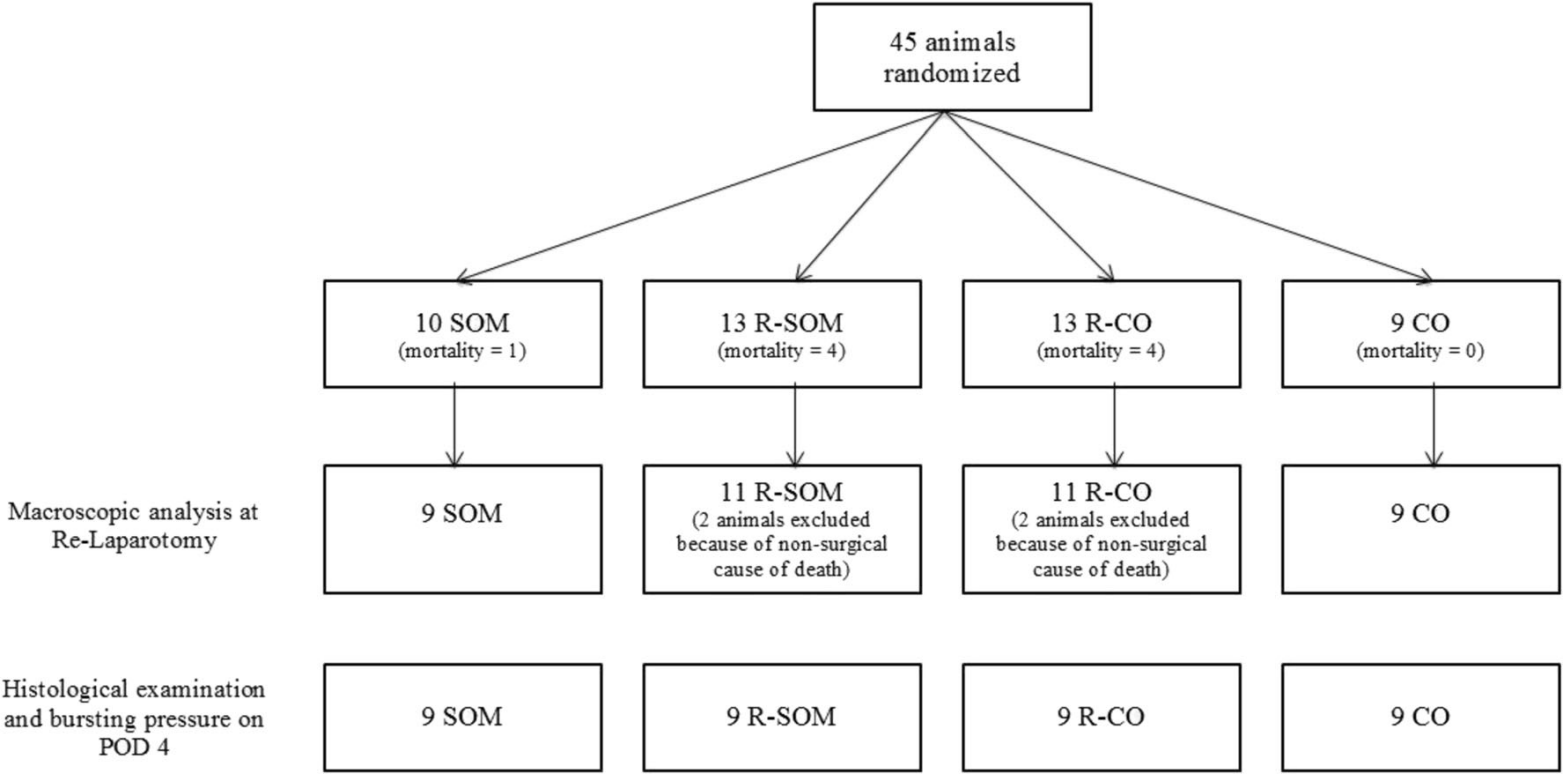
Flow chart of experimental protocol and mortality

In all animals, we observed significant postoperative weight loss by postoperative day 4 (POD 4) (*p* < 0.001 for all groups: Co − 22.4 ± 10.2 g; SOM − 20.1 ± 6.4 g; R-Co − 17.1 ± 11.2 g; R-SOM − 24.1 ± 6.8 g). There was no significant difference among the groups (*p* > 0.05). No animal died on the day of the operation. Total postoperative mortality was 20% for all groups (Fig. 2). All animals were autopsied and examined regarding most probable cause of death. None of the Co animals died. In the SOM group, one rat died on POD 2 (nearly half the anastomotic circumference was insufficient), resulting in a postoperative mortality of 5.3% for all non-irradiated animals. This is in contrast to a 30.8% mortality rate in both irradiated groups. In the R-Co group, two animals died on POD 1, and two died on POD 2. One of these animals suffered an anastomotic leak (nearly half the circumference was insufficient), one displayed ileal obstruction in the anastomotic region, one had peritonitis without signs of leakage, and one did not display any obvious intra-abdominal pathology. Of the R-SOM animals, one died on POD1, and three died on POD2. Two of these animals suffered ileal obstruction in the anastomotic region, and two had no obvious intra-abdominal pathology. Anastomoses from irradiated animals that died without observable direct cause of death were excluded from further evaluation.

To summarize, there was a significant difference in mortality between animals with and without irradiation treatment (*p* = 0.021), and Co animals differed both from R-Co (*p* = 0.046) and R-SOM animals (*p* = 0.046).

### Bursting pressure

**Fig. 3:**
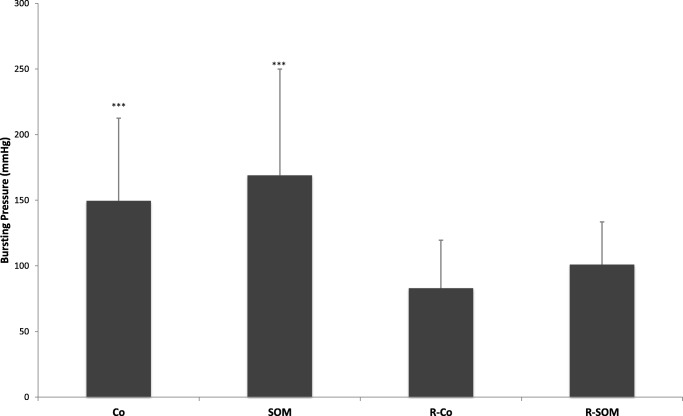
Anastomotic bursting pressure at re-laparotomy on POD 4. Mean values of irradiated and non-irradiated animals were significantly different (CO vs. R-CO, *p* = 0.004; CO vs. R-SOM, *p* = 0.009; SOM vs. R-CO, *p* = 0.006; SOM vs. R-SOM, *p* = 0.029). There was no statistically significant difference between the control groups and their respective pasireotide counterparts

Anastomoses were resected on POD 4, and bursting pressure was measured in 36 animals (mean values, Co 149.5 mmHg; SOM 169 mmHg; R-Co 83 mmHg; R-SOM 101 mmHg). Anastomoses of animals that died prematurely were excluded from analysis. In each case, the bursting site was located in the anastomosis. Mean values of irradiated and non-irradiated animals were significantly different (Co vs. R-Co, *p* = 0.004; Co vs. R-SOM, *p* = 0.009; SOM vs. R-Co, *p* = 0.006; SOM vs. R-SOM, *p* = 0.029). However, there was no significant difference between the control groups and their respective pasireotide counterparts (Fig. 3).

### Adhesions and anastomotic leakage

Adhesions at re-laparotomy were scored in 31 of 36 animals on POD 4 (86.1%). Both groups treated with pasireotide had lower adhesion scores than their respective controls with irradiation, but these differences were not statistically significant (*p* > 0.05). Interestingly, anastomotic leakage occurred in four cases, two from the SOM and two from the R-Co group. One animal of each group died prematurely, and leakage combined with generalized peritonitis was seen at autopsy. The other two cases were discovered at re-laparotomy on POD 4. The insufficiencies were covered by adhesions in these cases.

### Histology

**Fig. 4:**
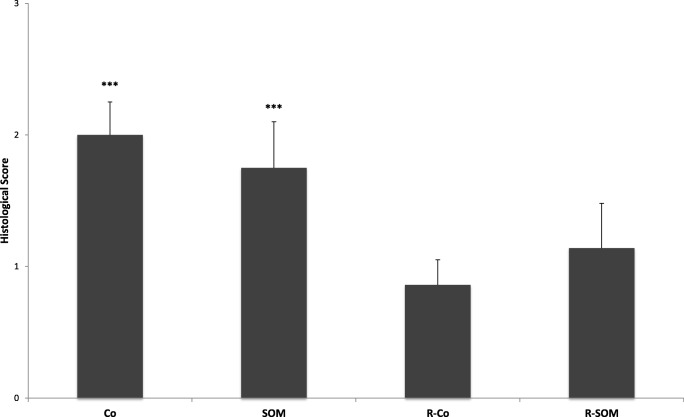
Granulocyte score on POD 4 (MV ± SD). The cell counts in the R-CO and R-SOM groups are significantly lower compared to the control group. SOM animals showed significantly more granulocyte invasion compared to the R-CO group. There were significant differences between the CO and the R-CO group (*p* = 0.001), the CO vs. the R-SOM group (*p* = 0.031), and the SOM vs. the R-CO group (*p* = 0.029)

Due to partial destruction during the bursting pressure test, some of the anastomoses could not be evaluated fully. Anastomotic sections were subjected to detailed analysis and scored according to the scoring system of Biert et al. [3]. There were no significant differences with respect to villus length, goblet cell count, areas of necrosis, extent of epithelialization, interstitial edema, submucosal fibroblast bridging of the anastomosis. R-Co animals appeared to have less bridging than other animals, but the difference did not reach significance. Collagen density of the anastomotic region was determined in sirius red–stained sections in a semi-quantitative fashion and appeared less in irradiated animals, but this too was not significant (Table 1). The cumulative scores of infiltrating granulocytes, lymphocytes, and macrophages were significantly lower in irradiated animals (*p* = 0.023). Granulocyte infiltration is illustrated in Fig. 4.

**Table 1:** Histological evaluation

Groups	Co		SOM		R-Co		R-SOM	
	M	SD	M	SD	M	SD	M	SD
Necrosis	1.33	1.22	1.37	0.92	1.71	1.25	1.17	0.75
Granulocyte*	2.00	0.50	1.75	0.71	0.86	0.38	1.14	0.69
Lymphocyte	1.11	0.60	1.25	0.46	1.29	0.49	1.00	0.58
Macrophage	1.56	0.53	1.63	0.52	1.43	0.98	1.14	0.38
Edema	0.78	0.67	0.86	0.69	0.71	0.49	0.83	0.41
Epithelium	2.00	0.87	2.14	0.69	2.14	0.69	2.00	0.63
Fibroblasts	1.38	0.74	1.38	0.92	1.86	0.90	1.40	0.55
Collagen	1.38	0.92	1.37	1.19	2.43	0.79	2.00	1.00

This table lists all histological anastomotic scores organized by parameter and group. *M* median, *SD* standard deviation. *Co* control group, *SOM* preoperative pasireotide injection, *R-Co* preoperative WBI, *R-SOM* preoperative pasireotide injection followed by WBI. * Only with regard to granulocytes were there significant differences between the groups

### Immunostaining and IGF-1

**Fig. 5:**
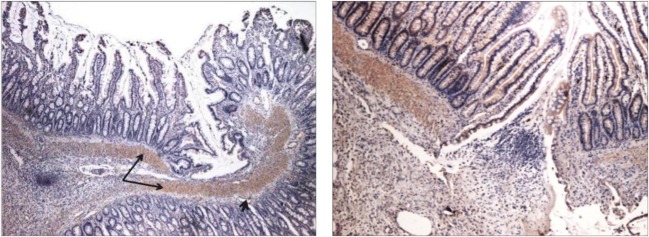
IGF-1 stain of anastomosis at 10-fold magnification. Control group (score = 2). The muscularis propria (long arrow) and muscularis mucosae as well as the endothelium (short arrow) stained intensely. Left: Co (score = 2) 10-fold magnification. Right: R-Co (score = 2) 10-fold magnification

Positive and negative controls were used to test for proper staining of IGF-1. Histological sections were scored. In all four groups, there was weak to moderate immunoreactivity (mean score ± SEM, Co 1.1 ± 0.9; SOM 1 ± 0.7; R-Co 1.3 ± 0.7; R-SOM 1 ± 0.8). The strongest staining was seen in endothelial cells, the muscularis mucosae, and the muscularis propria (Fig. 5). Granulation tissue showed very little staining. There were no significant differences among the groups. Both groups treated with pasireotide showed the least staining.

## Discussion

In radiation-induced mucosal injury, pancreatic enzymes appear to have a detrimental effect by further breaking down the intestinal barrier, autodigestion of extracellular matrix components, promotion of bacterial translocation, and induction of inflammatory processes [18, 24]. In previous studies by Fu et al., pasireotide, a somatostatine analogue with a long half-life and high-affinity receptor binding, was shown to mitigate radiation-induced mucosal injury, decreasing bacterial translocation and systemic inflammation. Furthermore, pasireotide administered prophylactically improved overall survival [8, 9]. Similar effects and a reduction of oxidative stress and inflammatory response were seen by Abbasoglu et al. [1]. Thus, large surface intestinal injury due to radiation appears to be vulnerable to pancreatic enzymes.

Our primary interest lies in intestinal anastomotic healing, a process that relies on specific and complex mechanisms. The anastomotic healing process is not as widely exposed to the action of pancreatic enzymes as is the case in diffuse irradiation injury. However, patients who receive neoadjuvant irradiation bear a considerably higher risk for postoperative anastomotic leakage than patients who do not [10, 17]. This is often ensued by complicated and protracted postoperative courses with development of pelvic abscesses, fistulas, and peritonitis. The effects of irradiation on anastomotic healing have been shown in animal experiments in the past [3, 7, 13]. We were interested in testing whether pasireotide, which is believed to decreases diffuse intestinal damage and systemic inflammation, also ameliorates intestinal anastomotic healing following irradiation.

Due to its receptor affinity profile, pasireotide has a broad spectrum of effects. Among others, it effectively decreases circulating GH and IGF-1 levels, which are both important for intestinal healing [2, 4–6, 15, 20, 27]. But on the other hand, GH has even been shown to improve anastomotic healing after irradiation [28]. Furthermore, in anastomotic healing, especially local production of IGF-1 appears to be beneficial [21, 29, 30].

By examining anastomotic healing under the influence of pasireotide both under control conditions and after irradiation, we expected to shed light on the relevance that these possibly contrary effects bear during anastomotic healing.

By measuring blood glucose levels after pasireotide administration, we tested for reliable drug delivery. Furthermore, a wide set of anastomotic quality parameters were determined to differentiate between the different groups of our trial.

## Postoperative complications

As expected, irradiation had a negative effect on perioperative outcome, mortality, and quality of anastomoses. We observed more complications and a high mortality rate of 30.8% in irradiated animals in both groups with and without pasireotide treatment. In three animals of the irradiated groups (23.1%), no obvious reason for death was found. Mortality due to small-bowel anastomosis-related causes is roughly 3.6% [16]. In a model of total-body irradiation, Vriesendorp et al. showed higher early mortality after adding irradiation of the abdomen that was most probably caused by the intestinal syndrome entailing watery diarrhea and lethargy [25]. This intestinal syndrome leads to a mean survival time of 5 days after irradiation. Our animals with undefined causes of deaths died on POD 1 or POD 2; this corresponds to 5 or 6 days after WBI, making radiation effects the most probable cause.

Pasireotide injection seems to have no negative influence on postoperative complications and mortality whereas animals with WBI show a much higher complication rate.

## Bursting pressure and IGF-1 levels

In both groups treated by radiation, anastomotic quality as measured by bursting pressure was considerably worse. This is reflected by the decrease in bursting pressure following irradiation. SOM treatment did not increase bursting pressure.

Despite its inhibiting effect on GH and IGF-1 levels, immunohistological exams showed no significant difference in local IGF-1 staining among these groups. Immunostaining for IGF-1 was weakest in the anastomotic region in both groups treated with pasireotide, but this difference was not statistically significant. This may be explained by local release mechanisms that bypass pasireotide dependent central nervous inhibition of the GH-IGF-1 axis. We have shown that a complex machinery of IGF-1, its receptors, and a large group of IGF-1 binding proteins is upregulated in the intestine during anastomotic healing [22]. It remains unclear how dependent this upregulation is of upstream hormonal stimulation and to which degree this is an autonomous, immune system–mediated effect. Data presented by Hao et al. indicate that local IGF-1 is at least partially independent of circulating GH [12].

A further inference that is corroborated by our data is that pancreatic enzymes do not significantly affect physiological anastomotic healing. This makes sense in as far as the interface between the healing process and pancreatic enzymes is very limited.

## Conclusion

Pasireotide does not prevent irradiation-induced impairment of anastomotic healing. Though the results of Fu et al. appear promising and seem to indicate a possible benefit of pasireotide not only for diffuse intestinal injury but also for anastomotic healing in the environment of the injured intestine, this cannot be supported by our data [9]. This may be due to a sufficient autonomous release system of IGF-1, the remoteness of the primarily submucosal anastomotic healing zone from pancreatic enzymatic action, and alternative mechanisms of irradiation injury.

Several critical points can be made. For one, pasireotide was given at a very high dose in our trial. This does not mirror clinical practice. However, it does not appear likely that lower doses would lead to different results. The most important remaining question in our view is whether results may have differed at later time points in the healing process. Anastomoses were examined on POD 4 and 8 days after irradiation. These time points are dominated by acute radiation-induced effects rather than long-term healing processes. We assume that examining anastomoses at later time points, e.g., 21 days may lead to different results [19].

In conclusion, our data show that pasireotide does not mitigate impaired anastomotic healing following irradiation. More importantly, our data present strong evidence that pasireotide does not negatively impact anastomotic healing.
